# Investigation of Flame Structures of Double-Base Propellant and Modified Double-Base Propellant Containing Nitramine Using OH-PLIF and Kinetic Simulation

**DOI:** 10.3390/molecules29051175

**Published:** 2024-03-06

**Authors:** Yiping Wang, Yan Zhang, Heng Li, Ergang Yao, Jin Yu, Fengqi Zhao, Siyu Xu

**Affiliations:** 1 National Key Laboratory of Energetic Materials (NKLEM), Xi’an Modern Chemistry Research Institute, Xi’an 710065, China; yip_wang@163.com (Y.W.); zhyan0529_901@163.com (Y.Z.); zerolhheart@163.com (H.L.); yaoerg@126.com (E.Y.); yujinnayo@163.com (J.Y.); npecc@163.com (F.Z.); 2 Shaanxi Laboratory of Energetic Materials, Xi’an Modern Chemistry Research Institute, Xi’an 710065, China

**Keywords:** combustion characteristics, double-base propellant, nitramine, OH-PLIF system, kinetic simulation

## Abstract

The combustion behavior of various propellant samples, including double-base propellants, pressed nitramine powders, and modified double-base propellants containing nitramine, was examined using OH-PLIF technology. The combustion process took place within a combustion chamber, and images capturing the flame at the moment of stable combustion were selected for further analysis. The distribution and production rate of OH radicals in both the double-base propellant and the nitramine-modified double-base propellant were simulated using Chemkin-17.0 software. The outcomes from both the experimental and simulation studies revealed that the concentration of OH radicals increased with a higher content of NG in the double-base propellant. In the modified double-base propellant containing RDX, the OH radical concentration decreased as the RDX content increased, with these tendencies of change aligning closely with the simulation results.

## 1. Introduction

High-energy and low-characteristic signals are the focus of the perpetual development of solid rocket propellant, which is widely used in modern military or space rocketry, such as composite modified double-base (CMDB) propellant. Double-base propellant containing nitrogen heterocyclic nitramines like cyclotrimethylenetrinitramine (RDX) and cyclotetramethylenetetranitramine (HMX) offers greater strength and more efficient combustion characteristics compared with traditional propellants. Elghafour et al. introduced a new variety of nitramine-based double-base propellants with up to 20 wt% RDX content, manufactured using solventless extrusion technology, and substantiated that the specific impulse (I_s_) was enhanced by 20% [1]. The nitramine particles and the decomposition gas of the double matrix (nitroglycerin(NG), nitrocellulose(NC)) diffuse and mix at the propellant combustion surface, forming a relatively homogeneous gas. The primary components of double-base propellant are nitrate (NG, NC), and the energy density can be expressed in terms of the number of -O-NO_2_ chemical bonds per unit mass of the propellant.

The steady-state combustion characteristics and theoretical model of these types of double-base propellants have garnered significant attention in the past few years [1,2,3,4,5,6]. Volkov and Paletsky et al. observed a two-zone structure in the nitramine flame at pressures of 0.1 MPa and 1 MPa. The consumption of nitramine vapor, partial consumption of NO_2,_ and formation of CH_2_O, HCN, NO, N_2_, etc. were detected in the flame structure using molecular beam mass spectrometry [7,8]. Yan et al. systematically investigated the detailed thermal decomposition mechanisms of the chemical reactions in different combustion zones of the nitramine-based double-base propellant. The oxidation mechanisms of NO_2_, formaldehyde (CH_2_O), and hydrogen cyanide (HCN) are predominantly involved in the combustion mechanisms [3,9]. Experimental and numerical simulations were used to explore the detailed combustion wave and distribution of temperature over the condensed phase, while also effectively predicting the sensitivity of burning rates at various temperatures and pressures. However, there are limited experimental data regarding the gas phase combustion reaction at the microscopic scale of the CMDB propellant containing nitramine as RDX.

Planar laser-induced fluorescence(PLIF), as a real-time, high-speed, two-dimensional flame diagnosis technique compared with traditional physical probes and sampling measurement, has been extensively utilized in the combustion diagnosis of energetic materials. The PLIF technology can not only support 2D imaging of important intermediates (such as OH, NO, CH_2_O, etc.) or their concentration but also the flame structure during the combustion process. Parr et al. conducted measurements of the two-dimensional NH, OH, and CN species profiles for ADN, AP, HNF, GAP, etc. using PLIF and discussed the flame structure of the solid propellant [10,11]. Ruesch et al. utilized PLIF technology to investigate the distribution of CN and OH during the combustion of cocrystals of CL-20 and a polycrystalline composite crystal of HMX/AP. The variation in burning rate was explained by the discrepancy in flame structure [12]. Chevalier et al. carried out combustion experiments of aluminum-containing solid propellant under a pressure of 1.0 MPa. The aluminum vapor generated was detected by high-speed Al-LIF to simulate the concentration and temperature distribution of aluminum atoms around the combustion of aluminum droplets through a one-dimensional quasi-steady-state model [13]. These experiments demonstrate that PLIF is an effective online diagnostic method for combustion. In the combustion of hydrocarbon fuels, OH radicals serve as a crucial intermediate in the majority of radical reactions and are widely used to delineate the combustion reaction zone. They play a significant role in indicating the location of the flame front and defining the combustion characteristics of the flame. In addition, OH radicals have the characteristics of higher concentration, stronger absorption, and a distinctive radiation spectrum in the UV band, which facilitates their easy detection. Consequently, OH radicals are some of the most important and common measurement objects in PLIF technology. Xin et al. evaluated combustion deterioration in kerosene spray flames through the use of a simple OH-PLIF technique [14]. Yang et al. studied the change in flame height and area of insensitive triple-base propellants over time using OH-PLIF technology and found that the high concentration of OH radicals is mainly located outside NO, suggesting that there may be a transformation between NO and OH radicals. They indicated that both the concentration of the desensitizers and the methods of desensitization have important effects on the reaction process [15]. Yan et al. used a high-speed camera and OH-PLIF to capture the luminescence characteristics and OH concentration distribution in the flames of TKX-50 and TKX-50/AP composites. They found that preignition occurred twice when the AP materials were added and the peaks of the OH concentration signals were similar [16]. In summary, several OH-PLIF applications in recent years have shown that OH-PLIF plays an important role in exploring the changes in the intermediates of combustion reactions, helping researchers to discover changes in the state of combustion and revealing the detailed reaction mechanisms [17,18,19].

The major objective of this research was to examine the chemical flame structures (distributions of OH radicals) of double-base propellant, cyclic nitramine (RDX), and modified double-base propellant with RDX during their combustion at a pressure of 0.1 MPa. The investigation also encompassed analyzing the influence of varying NC concentrations within double-base propellants, and the effects of altering the content of RDX in modified double-base propellants with nitramine on the distribution of OH radicals were also considered. Additionally, The effect of NG or RDX addition on the chemical properties was also evaluated using CHEMKIN-17.0 software [20]. This study utilized established combustion models to simulate the combustion processes of the propellants, with the aim of elucidating the underlying principles and outlining the detailed reaction mechanisms during combustion.

## 2. Results and Discussion

### 2.1. OH-PLIF Signals of Propellants

#### 2.1.1. Double-Base Propellant

**Figure 1:**
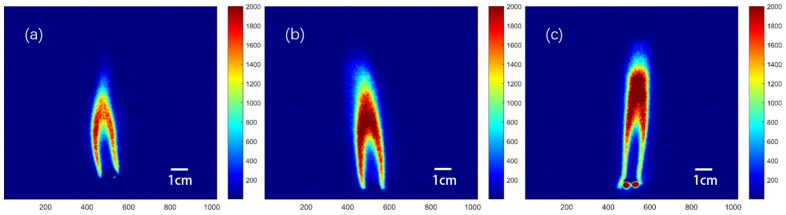
OH-PLIF image of combustion of double-base propellant at 1 atm. (**a**) DB1 with 16% NG content, (**b**) DB2 with 26% NG content, and (**c**) DB3 with 36% NG content.

The OH radical serves as an important intermediate in the combustion process, responding to the burning intensity and structure of the flame. Our research in this study emphasizes the dark zone and the luminous flame region. Before investigating the effect of nitramine addition on the combustion of double-base propellants, we conducted a study of the combustion of double-base propellants with different nitroglycerine concentrations. Redox reactions occur between reaction products mainly in the dark zone. These reactions are slow and can be accelerated at high temperatures and pressures. The luminous flame region, situated at the end of the flame in the dark zone, generates final products and reaches thermal equilibrium. The OH-PLIF image in Figure 1 shows the combustion of double-base propellant with different NG concentrations (16%, 26%, and 36%) at 1 atm, and typical laminar pre-mixed flame structures can be observed. The OH-PLIF signals are predominantly distributed on both sides and above the middle of the flame. An increase in the NG ratio enhances the concentration of OH-PLIF signals, indicating significant strengthening of the flame’s combustion intensity [21]. Signal strength maxima move in a higher direction. Signals below 10% of the maximum strength were filtered, and those above the threshold were analyzed as effective signals.

**Figure 2:**
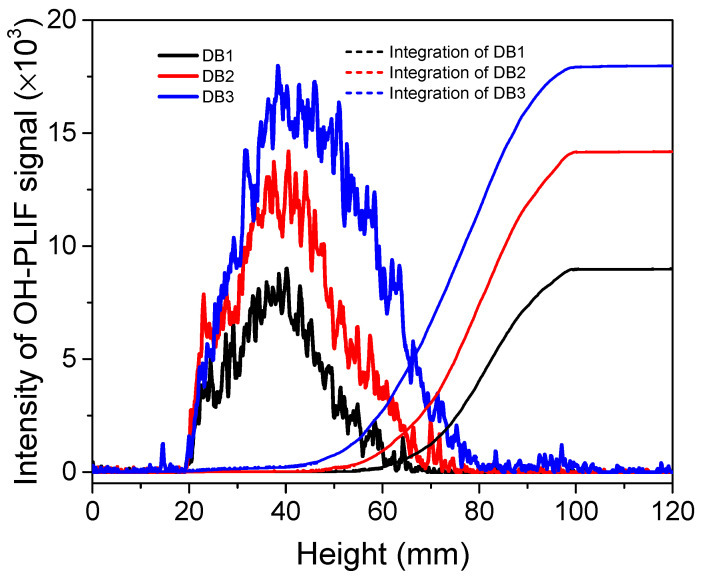
Transverse signal strength integration along the flame height and global integration.

Figure 2 illustrated the transverse integration of effective signal strength across flame height and globally. Differences in signal strength and distribution heights are more evident in the integration plot. The heights of the OH-PLIF effective signal distributions for the three types of propellants are 42.2 mm, 46.5 mm, and 52.1 mm, respectively, indicating a trend of increasing flame height with higher NG content and the generation of more OH radicals. NC exists as a long-chain polymer with three potential nitration sites on each monomer. Military NC typically contains 12–13% N and 57–59% O, while NG contains around 63.44% O. The results of the PLIF experiment corresponded to an increased concentration of OH radicals due to the higher oxygen content. In the case of double-base propellants, the initial reaction on the burning surface is generally considered as follows,

NG → NO_2_ + H_2_O + vapor (R1)

NC → CH_2_O + NO + NO_2_ + CO + CO_2_ + H_2_O + HCOOH (R2)

NO_2_ acts as an oxidizer, while CH_2_O acts as a fuel component. NO_2_ and CH_2_O react rapidly to form carbon oxides and nitrogen oxides, generating OH radicals in the process. The reactions are generally considered to be as follows:

NO_2_ + CH_2_O → NO + H_2_O + CO (R3)

2NO_2_ + CH_2_O → 2NO + H_2_O + CO_2_ (R4)

NO_2_ molecules are almost completely consumed in this reaction zone. In the later luminous flame zone, nitric oxide produced in the dark zone generates OH radicals with free radicals such as H, NH_2_, or H_2_O [22]. The specific reaction equations are as follows:

H + NO + H(+M) → NH + OH(+M)(R5)

NO + NH_2_ → N_2_ + H + OH (R6)

NO(+M) + H_2_O → NO + H + OH(+M) (R7)

**Figure 3:**
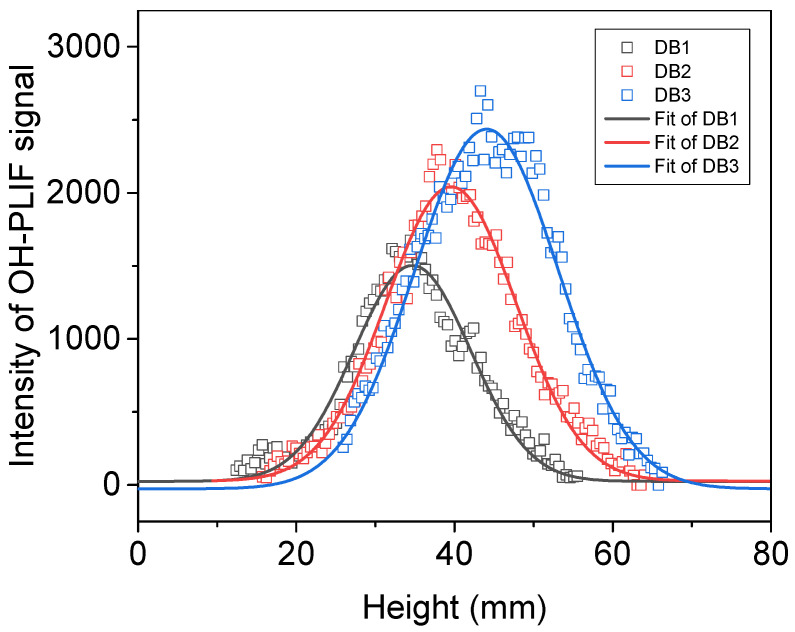
Distribution of the OH-PLIF signal in the axial direction at the center of the flame of double-base propellant.

Along the central axis of the flame, a line is drawn to investigate the change in signal strength with height. This approach allows for subsequent cross-referencing with simulation results. A second-order Gaussian function is fitted to the experimental data points distributed along the axial direction of the flame’s surface. The distance between the peak of the gradient and when the gradient reaches zero is considered to be the flame reaction brush thickness (δ_OH_), which is a novel definition [23] (see Figure 3). This definition serves as a standard for measuring changes in the reaction zone (OH radicals). The reason for only considering the one-dimensional axial distance from the peak of the rising intensity gradient to the intensity maximum is that the impact of heat dissipation on the combustion reaction is not accounted for in one-dimensional laminar flames of CHEMKIN simulations, leading to simulated concentrations not decreasing. Consequently, the peak of the falling gradient cannot be obtained, making it an ineffective way to illustrate the correlation between experimental results and simulations. Subsequently, this method was employed to expand the investigation to other propellants and facilitate the comparison of experimental data with simulation results.

For the three samples DB1, DB2, and DB3, the flame brush thicknesses measured were 6.25 mm, 7.96 mm, and 9.13 mm, respectively. The flame brush thickness tends to increase with the rising NG contents. Additionally, the peak intensity of the OH signal also increased with increasing NG contents, and the peak moved further away from the burning surface. This indicates that the heights of the luminous flame regions were elevated, and the combustion reaction became more intense with the addition of NG [8].

#### 2.1.2. RDX Specimen

**Figure 4:**
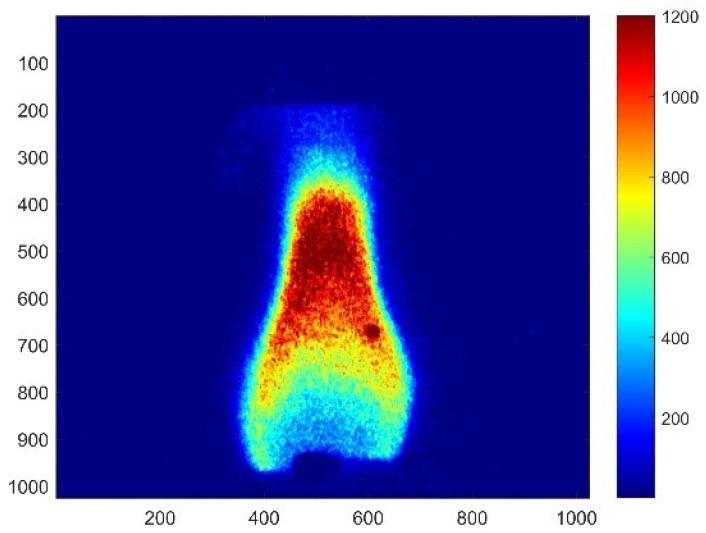
OH-PLIF image of combustion of RDX at 1 atm.

**Figure 5:**
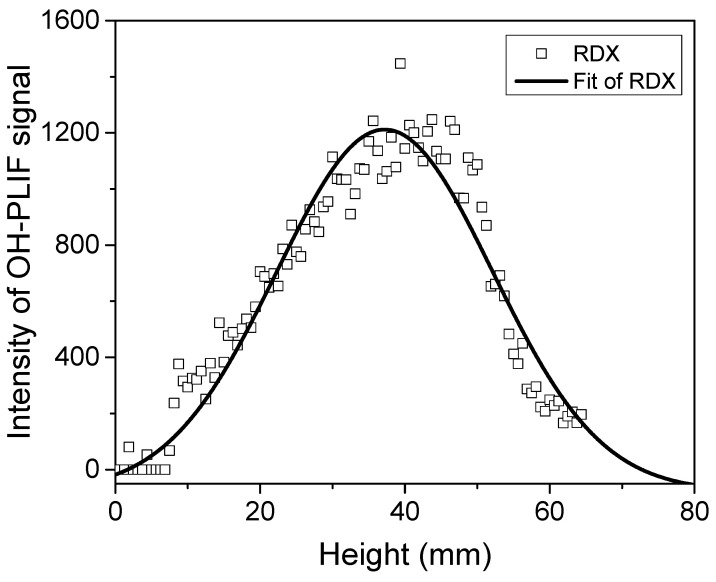
Distribution of the OH-PLIF signal in the axial direction at the center of the flame of RDX.

In the experiment, RDX powders were compacted into 5 mm diameter and 6–8 mm height strands using a tablet press. The OH-PLIF signal structure of RDX combustion at 1 atm is shown in Figure 4. A flame with distinct dark zones and luminous flame zones can be seen. RDX burns relatively steadily with a certain frequency pulsation. This experimental phenomenon is consistent with the observations of Volkov et al. [7] in their observation of RDX combustion. They pointed out that the formation of large bubbles was observed on the surfaces of strands in all experiments. In some cases, these bubbles completely disappeared after ignition, and in others, they remained on the strand surface during combustion. When bubbles were present on the burning surface, the combustion of RDX strands was unstable and even ceased several seconds after ignition. In a significant number of cases (~40% of all experiments), stable combustion (without bubbles) was observed with a burning rate of ~0.27 mm/s. The moments when the flame pattern of the substance was nearly identical were recorded for further study. OH radicals were distributed above the luminous flame zone, and no high concentrations of OH radicals were found on either side of the dark zone in the combustion flame of nitramine compared with the double-base propellants. The distribution trends of OH radicals during the combustion process were similar for double-base propellant with peak concentrations at 31.2 mm height to the surface of combustion, as shown in Figure 5. Additionally, The flame brush thickness was 20.65 mm for the samples of RDX, which indicates that the high mole fraction of OH radicals is closer to the surface of combustion in nitramine than in double-base propellant.

Combustion of RDX was reported in several studies [6,24] that used mass-spectrometry techniques (including gas sampling) and various advanced optical techniques. The variation trend of the experiment is in general agreement with the results of the PLIF test on RDX by Parr et al. [25]. Brutto reactions of the transformation of RDX in final combustion products (nitramine → ΣαiAi) based on modified compositions can be expressed as [7]

RDX → 2.77 N_2_ + 2.27 CO + 1.79 H_2_O + 0.93 H_2_ + 0.64 CO_2_ + 0.47 NO + 0.36 H + 0.25 OH (R8)

The shortage of H in the final combustion product compositions, in comparison with the initial element compositions, is primarily attributed to the absence of H and OH radicals in these compositions. The high concentrations of H and OH radicals are involved in the thermodynamic equilibrium components. In order to calculate temperature so that the corresponding composition of the final combustion products can be measured, it is necessary to add the H and OH radicals in the Brutto reactions. In the first flame zone, the consumption of NO_2_ and CH_2_O with the formation of H_2_, H_2_O, CO, N_2_, CO_2_, HCN, and NO occurs, while a small amount of OH radicals is generated. The main reaction is the oxidation of HCN by NO accompanied by the formation of final combustion products in the second flame zone. The significant production of OH radicals during this process is observable in the luminous zone using PLIF.

#### 2.1.3. Double-Base Propellant Containing RDX

**Figure 6:**
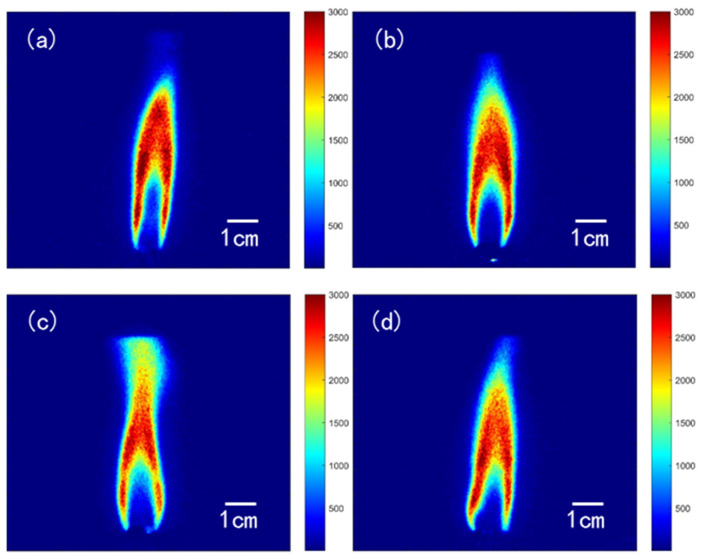
OH-PLIF image of combustion of MDB propellant at 1 atm. (**a**) MDB1 with 6% RDX content, (**b**) MDB2 with 16% RDX content, (**c**) MDB3 with 26% NG content, and (**d**) MDB3 with 26% NG content.

The distribution of OH-PLIF signal in nitramine-MDB propellants resembles that of double-base propellants. The gas phase can be divided into a dark zone and a luminous flame zone. Typical OH-PLIF signal structures of double-base propellant containing nitrogen heterocyclic nitramines are shown in Figure 6 as a function of percentage composition. A luminous flame zone is observed at a distance from the burning surface, generating high OH concentrations above it. As RDX contents increase, the zone of high OH concentration moves slightly closer to the burning surface. Moreover, the OH-PLIF signal follows an increasing–decreasing pattern with axial height, with the flame eventually forming a conical shape downstream. Miller et al. [4] pointed out that

HNO + NO = N_2_O + OH (R9)

is the primary source reaction for radical buildup in the case of nitrate esters. Several paths of generation of OH, including reactions between NO, HNO, and NH with H radicals, NH_2_ radicals, and H_2_O, can contribute to radical buildup.

**Figure 7:**
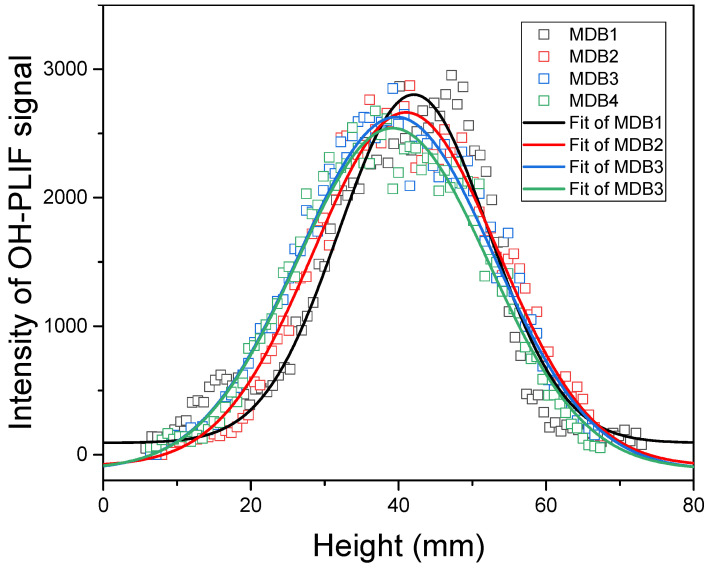
Distribution of the OH-PLIF signal in the axial direction at the center of the flame of MDB propellant.

The OH-signal map in the axial direction at the center of the flame and a comparison of the different specimens are shown in Figure 7. The maximum value of the OH signal for MDB1 in the figure is about 3372, while for MDB2, MDB3, and MDB4, the maximum values are 2984, 2906, and 2591, respectively. The fitted curve discarded some points with larger values, yet the overall trend distribution does not change significantly. The fluorescence signal intensity indicates that as the RDX content in the propellant samples increases, the maximum OH signal intensity decreases. The maximum OH signal for the four samples is observed at approximately the same distance, around 3 cm from the burning surface. Based on derivative calculations, the brush thicknesses are 7.96 mm, 10.70 mm, 12.03 mm, and 16.79 mm. Flame brush thickness appears to decrease as the RDX content increases. This indicates that the height of the luminous flame regions is elevated and the combustion reaction is more intense with the addition of RDX. The decrease in OH signal intensity may be attributed to the lower mass fraction of oxygen in RDX compared with NC.

### 2.2. Detailed Mechanism Analysis Using CHEMKIN

The combustion characteristics between double-base propellant and nitramine MDB-propellants show a noticeable difference, particularly when considering the concentrations of NC and RDX. To clarify the reaction mechanisms of the different species of propellants, the premixed flames of specimens were simulated using the laminar premixed flame program in CHEMKIN [20]. The gas-phase input data for solid propellants refers to Miller [4] and Yetter [22] et al., as mentioned in this study.

**Figure 8:**
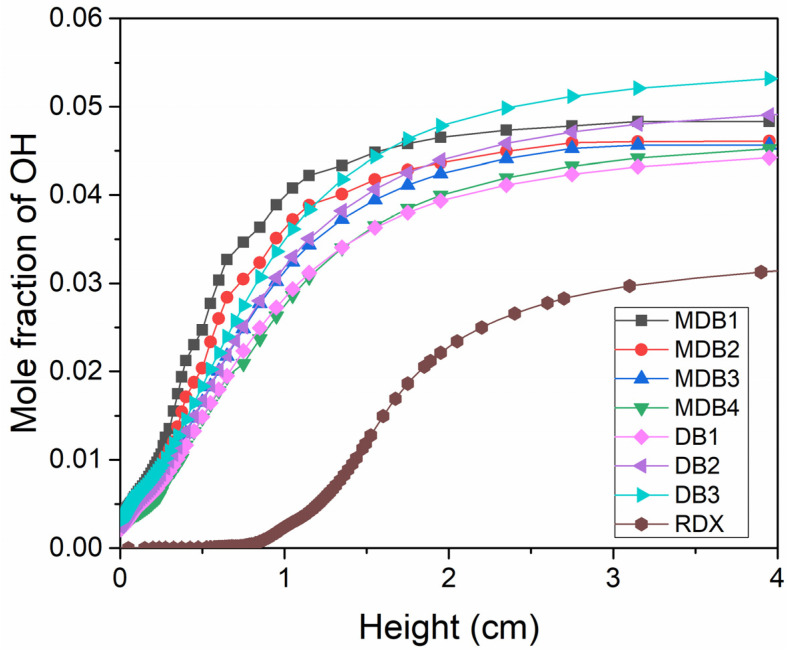
Simulation of the mole fraction of OH for the samples.

The simulation does not account for heat dissipation, resulting in the OH mole fraction not exhibiting a decreasing trend after reaching its peak value. Only the process of generation of OH radicals in the flame was considered during analysis of the data before the peak simulation results. The profile of the OH mole fractions of the samples, as a researched chain-branching species, is depicted in Figure 8. The results indicate that the mole fractions of OH decrease with increasing RDX in the nitramine-MBD propellant, while they increase with increasing NC in the double-base propellant. This observation is consistent with the results of the experiment. As one of the most important immediate species, the increase in the OH mole fraction can accelerate the flame propagation process. Moreover, a substantial amount of OH is observed at the luminous flame zone, in which the mole fractions of OH increase rapidly and reach their peak. The broader distribution of OH radicals in the flame of MDB propellants, compared with double-base propellants, is due to the incorporation of nitramine, enabling a more rapid combustion reaction and the earlier generation of OH radicals in the flame. The simulation results were derived and OH flame brush thicknesses were calculated using the method described above. The OH brush thicknesses were 20.53 mm, 25.70 mm, 31.32 mm, and 33.85 mm in the flames of the nitramine-MDB propellant from MDB1 to MDB4, respectively, and 23.86 mm, 29.72 mm, and 35.39 mm in the flames of the double-base propellant from DB1 to DB3. This trend is also consistent with the experimental results. The distribution of OH radicals produced by nitramine combustion is influenced by the powder particle size and compactness during the experiment, as OH radicals appear earlier than in the simulation results. Moreover, the combustion process of nitramine powder is not purely premixed flame combustion. Nevertheless, the simulation results are similar to the experimental outcomes in terms of OH flame brush thickness and the peak of OH concentration.

**Figure 9:**
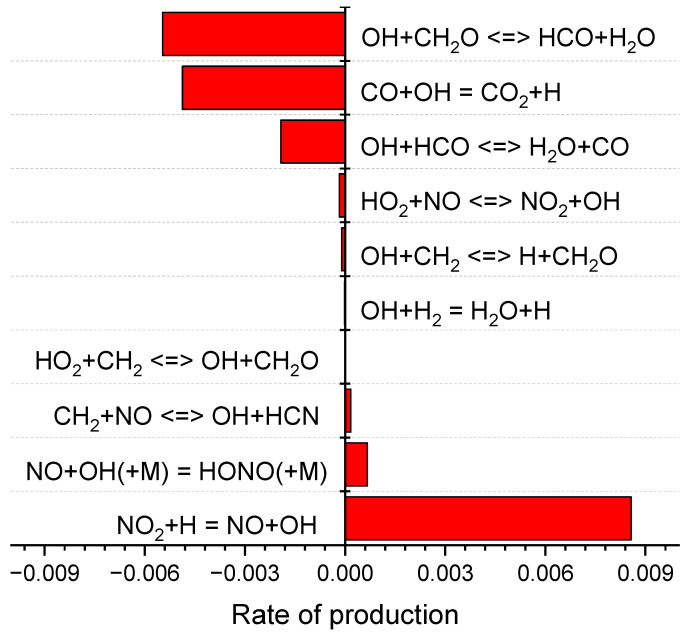
Rate of production (OH) of double-base propellant.

**Figure 10:**
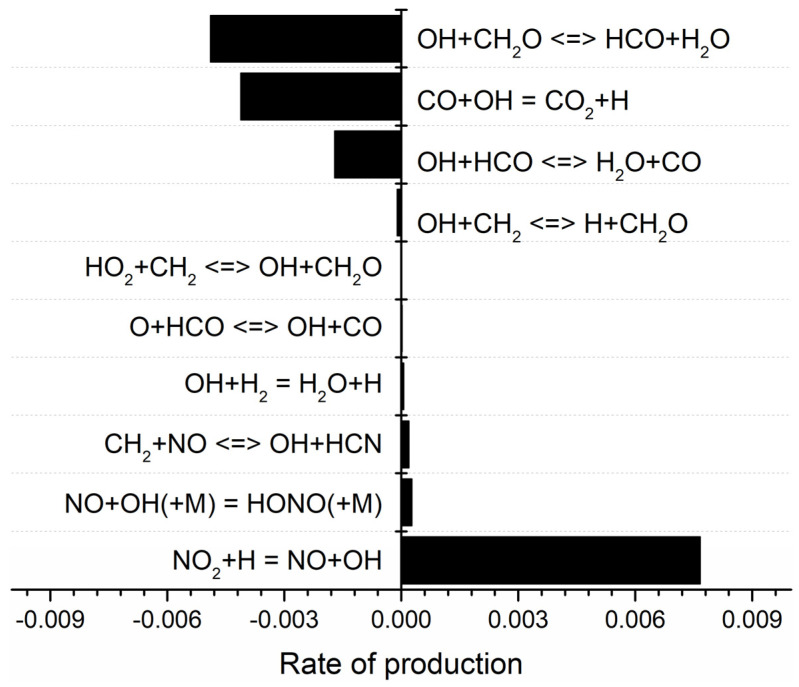
Rate of production (OH) of MDB propellant with RDX.

The rate of production (ROP) of OH was calculated with and without RDX. As shown in Figure 9 and Figure 10, the main reaction for the generation of OH radicals in both the presence and absence of RDX is

NO_2_ + H = NO +OH (R10)

while the reaction with the highest rate of OH radical consumption is

OH +CH_2_O ↔ HCO +H_2_O (R11)

The rate value of the two reactions decreased by 0.07% and increased by 3.18% when RDX was added. The peak of the total rate of OH radical generation decreased from 8.99 × 10^−3^ mol cm^−3^ s to 8.18 × 10^−3^ mol cm^−3^ s, and the total consumption rate increased from −1.19 × 10^−2^ mol cm^−3^ s to −1.08 × 10^−2^ mol cm^−3^ s. As an important chain-branched radical, OH exhibited a smaller decrease with the increase of RDX, which follows the experimental results. The simulation results indicate that NO_2_ plays a significant role in positively affecting OH generation. Anderson [26] and Cornell et al. [27] showed that the peak of the NO_2_ mole fraction produced by the burning surface of RDX at 1 atm ranged from 0.02 mol cm^−3^ s to 0.05 mol cm^−3^ s. The study of Glorian et al. [28] on NG showed that the peak of the NO_2_ mole fraction produced by the burning surface of NG during combustion was around 0.2 mol cm^−3^ s. The substitution of RDX for NG in the propellant leads to lower NO_2_ production at the combustion surface, which in turn results in a lower OH production rate. The results of the OH-PLIF experiment validate this simulation data well from a qualitative point of view.

## 3. Materials and Methods

### 3.1. Materials and Specimens

Cyclotrimethylenetrintramine (RDX), nitrocotton (NC), nitroglycerin (NG), dibutyl phthalate (DBP), dimethyldiphenylurea (C_2_), and vaseline (V) are used as components of composite modified double-base propellant. The following Table 1 presents the ratios of these materials in seven different propellants provided by Xi’an Modern Chemistry Research Institute.

### 3.2. Equipment and Experimentation

#### 3.2.1. Combustion and Ignition Systems

A pressure vessel with four fused silica windows on all sides was used as a combustion chamber. Two of the windows allowed the UV beam to enter and exit the pressure vessel, while a third window perpendicular to the laser sheet allowed imaging of the induced fluorescence signal. The vessel had connecting pipes above and below for gas input or output. The ignition device for the propellants was a nickel-chromium alloy wire, which was connected to a power source.

#### 3.2.2. PLIF System

**Figure 11:**
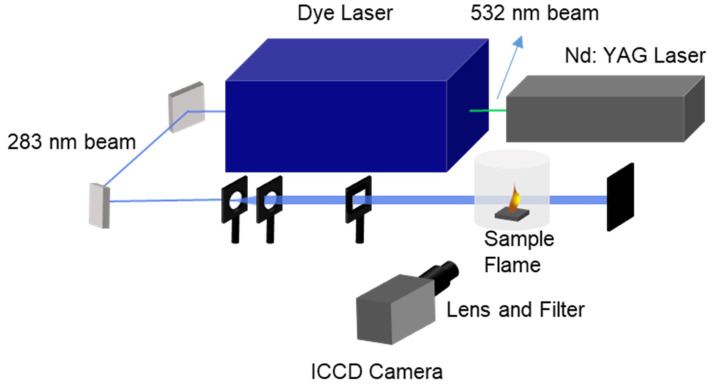
Diagram of PLIF system.

The experimental system in this study is shown in Figure 11, which includes a pulsed Nd: YAG 980 series laser (YG981E-10, Quantel, France) that produces a 1064-nm beam, 532-nm beam, or 355-nm beam for pumping a tunable dye laser. The flash lamp and Q-switch of the Nd: YAG laser are triggered continuously to generate laser pulses at a repetition frequency of 10 Hz. The frequency-doubled pulse power at maximum is 212 mJ, as measured. The beam is used to pump the dye laser (Q-SCAN, Quantel, France), where Rhodamine 590 dye mixed in ethanol is circulated through the oscillator and amplifier. The dye concentrations in the oscillator and amplifier are 0.07 g/L and 0.0175/L respectively. The output wavelength of the beam exiting the amplifier stage is 566.54 nm, which is then focused into a frequency-doubling crystal. The beam is isolated by a four-prism separator. The beam power is measured to be 13 mJ after frequency doubling. The Q_1_(7) line at 283.2 nm is excited by the adjusted beam.

The laser beam is expanded using a negative cylindrical mirror (f = −75 mm, Thorlabs, Newton, NJ, USA). Two positive cylindrical lenses (f = 100 mm, f = 499 mm, Thorlabs, Newton, NJ, USA) are then used to focus the expanded beam both vertically and horizontally. The OH fluorescence signal is detected by an ICCD camera, which comprises an optical filter (307FS10-50, Andover, UK) to remove interference signals. The gate of the ICCD camera is set to 100 nm and the delay is 50 ns to lessen the effects of ambient lights. In this experiment, all the images taken by the PLIF system are processed using background noise subtraction and median filtering. The screen resolution is set to 1024 × 1024, while the screen size is 80 mm × 80 mm for the sake of convenience to measure flame dimensions.

## 4. Conclusions

The combustion characteristics of double-base propellant, RDX powder, and nitramine double-base propellant were investigated using the OH-PLIF technique. Subsequently, ANSYS Chemkin17.0 was employed to model the combustion process of each sample, including an analysis of the OH mole fraction and production. The main conclusions can be summarized as follows:

(1) In the combustion flame of double-base propellant, OH radicals are predominantly found on both sides and at the downstream center. As the content of NG increases, the maximum signal intensity and overall signal intensity of OH-PLIF also rise, indicating an increase in the concentration of OH radicals. The OH flame brush was utilized to compare the trends in experimental and modeled OH radical generation accumulation values. This observation supports the predictions made by Yetter about the M2 and M9 double-base propellant models.
(2) OH-PLIF experiments were performed to analyze the combustion behavior of RDX powder-pressed pillars and modified double-base propellants containing RDX. Observations revealed that during the combustion of RDX powder, OH radicals were mainly concentrated at the central region of the flame’s distal end. In contrast, in the case of modified double-base propellant containing RDX, OH radicals were predominantly located along the flanks of the luminous flame zone and at the core of the distal flame end, displaying a distinct distribution pattern for OH.
(3) As the RDX content in the modified double-base propellant increased, a reduction in the generation of OH radicals was noted. This phenomenon was hypothesized, drawing on ROP analyses, to potentially stem from a concomitant rise in RDX concentration and a decline in the NG component. The combustion of NG yields higher production of NO_2_ compared with RDX, and NO_2_ plays a pivotal role as a reactant in the generation of OH radicals.

## Figures and Tables

**Table 1:** Contents of NC, NG, RDX, HMX, DBP, C2, and V in seven different propellant specimens.

Samples	Percent of Component/%					
	NC (12.0)	NG	RDX	DBP	C_2_	V
MDB1	49.52	36.48	6.00	5.00	2.50	0.50
MDB2	43.76	32.24	16.00	5.00	2.50	0.50
MDB3	38.00	28.00	26.00	5.00	2.50	0.50
MDB4	32.24	23.76	36.00	5.00	2.50	0.50
DB1	72.00	20.00	0	5.00	2.50	0.50
DB2	62.00	30.00	0	5.00	2.50	0.50
DB3	52.00	40.00	0	5.00	2.50	0.50

## Data Availability

Due to the nature of this research, participants of this study did not agree for their data to be shared publicly, so supporting data is not available.
